# Removal of Aromatic Chlorinated Pesticides from Aqueous Solution Using β-Cyclodextrin Polymers Decorated with Fe_3_O_4_ Nanoparticles

**DOI:** 10.3390/polym10091038

**Published:** 2018-09-19

**Authors:** Sebastián Salazar, Daniel Guerra, Nicolás Yutronic, Paul Jara

**Affiliations:** Department of Chemistry, Facultad de Ciencias, Universidad de Chile, Las Palmeras 3425, Ñuñoa, Santiago 7800024, Chile; sebasalazar@ug.uchile.cl (S.S.); nyutroni@uchile.cl (N.Y.)

**Keywords:** β-cyclodextrin, cyclodextrin nanosponges, water treatment, adsorption, nano sorbents, magnetic nanoparticles

## Abstract

This article describes the sorption properties of cyclodextrin polymers (nanosponges; NS) with the pesticides 4-chlorophenoxyacetic acid (4-CPA) and 2,3,4,6-tetrachlorophenol (TCF), including an evaluation of its efficiency and a comparison with other materials, such as granulated activated carbon (GAC). NS-pesticide complexes were characterized by scanning electron microscopy (SEM), energy dispersive spectroscopy (EDS), X-ray powder diffraction (XRPD), proton nuclear magnetic resonance (^1^H-NMR), UV–VIS, and thermogravimetric analysis (TGA). This confirms the interactions of the guests with nanosponges and shows that the polymers have favorable sorption capacities for chlorinated aromatic guests. Our studies also show that the inclusion complex is predominantly favored for NS/CPA rather than those formed between TCF and NS due to the size of the adsorbate and steric effects. Sorption studies carried with repeated cycles demonstrate that NS polymers could be an improved technology for pollutant removal from aquatic environments, as they are very efficient and reusable materials. Our experiments and characterization by SEM, EDS, UV–VIS, and magnetization saturation (VSM) also show that NS is an optimal substrate for the deposition of magnetite nanoparticles, thus improving the usefulness and properties of the polymer, as the nanosponges could be retrieved from aqueous solution with a neodymium magnet without losing its efficiency as a pesticide sorbent.

## 1. Introduction

The water quality around the world is deteriorating due to pollution caused by organic and inorganic compounds generated from anthropogenic activity. As a result of these activities, organic compounds, such as pesticides, are widely present in potable water sources. They are known to be extremely toxic and harmful to human health. Pesticides are chemical compounds that are applied extensively around the world to kill pests, including rodents, fungi, insects, and weeds. Pesticides are not only used in agricultural fields, but also in homes, parks, schools, buildings, and forests [1,2]. In addition, pesticides can be found in the air we breathe, the food we eat, and the water we drink.

The use of chlorinated aromatic compounds as pesticides, such as 4-chlorophenoxyacetic acid (4-CPA) and 2,3,4,6-tetrachlorophenol (TCF), has been approved by the Environmental Protection Agency (EPA) due to their moderate toxicity levels. Nonetheless, toxicological studies suggest that these pesticides cause health issues, such as ocular irritation and weight loss.

Higher exposures can cause lung irritation, shortness of breath, pulmonary edema, and anemia due to blood cell damage [3,4]. Extensive use of pesticides can also cause serious pollution to soil and groundwater.

Over the past few years, advances in nanotechnology adsorption have been achieved, as many conventional water treatment techniques (ion exchange, molecular sieves, coagulation, chlorination, ozonation, etc.) have limitations associated with their high cost, low efficiency, and residue generation [1,2,5]. β-Cyclodextrin (β-CD) nanosponges have been reported to be a great tool for the removal of pollutants from water [5,6,7,8,9]. CDs are water-soluble oligosaccharides, most commonly composed of six, seven, or eight glucopyranose units linked by α-1,4-glycosidic bonds.

Among the CD polymers, β-CD is interesting due to its size (7.8 Å with an average inner diameter of 7.8 Å), which can suitably accommodate benzyl compounds [6,7,8]. CDs have been used to form nanoporous materials because of their well-defined structure, moderate toxicity when administered orally/locally, versatility, and ability to form inclusion complexes [6,7,8,9,10,11].

Magnetite nanoparticles have also attracted considerable attention because of their many potential applications. As one of the most common applications, superparamagnetic nanoparticles are known to be great sorbent materials, as they are extremely easy to handle by imposing external magnetic fields. However, superparamagnetic nanoparticles, such as magnetite, have several limitations, such as a lack of molecular selectivity, low binding capacity to target molecules, and aggregation, to name a few [12,13,14,15]. Depositing magnetic nanoparticles at the surface of a polymer might maintain the advantages of superparamagnetic particles while allowing these shortcomings to be overcome.

The objectives of this study were to synthesize and characterize β-CD polymers (nanosponges; NS) decorated with Fe_3_O_4_ nanoparticles and to investigate the sorption properties of NS with a series of pesticides (4-CPA and TCF), as these polymers have been reported to have great potential for the sequestration of chlorinated aromatic compounds from aqueous media to achieve relatively low concentration levels.

The sorption properties of NS were compared with those of granular activated carbon (GAC). This study contributes to the understanding of the potential utility of NS for the removal of pesticides from aqueous environments.

## 2. Methods

### 2.1. Materials

All chemical reactants used in this study are commercially available and were used as received: β-cyclodextrin (Sigma-Aldrich, Saint Louis, MO, USA), 4-chlorophenoxyacetic acid (Sigma-Aldrich), 2,3,4,6-tetrachlorophenol (Sigma-Aldrich), diphenylcarbonate (Sigma-Aldrich) and miliQ water (Merck, Darmstadt, Germany). Glassware used for the experiments was washed with aqua regia (molar ratio of 3:1 between HCl and HNO_3_) and then rinsed repeatedly with milli-Q water.

### 2.2. Synthesis of Fe_3_O_4_ Nanoparticles

Fe_3_O_4_ nanoparticles were synthetized by the co-precipitation method, as reported previously [13]. Solutions of 0.1 M FeCl_2_·4H_2_O (1 mL) and 0.2 M FeCl_3_·6H_2_O (4 mL) were prepared in 0.1 M HCl in a round bottom flask under argon gas supply to maintain inert atmosphere. Precipitation was performed with dropwise addition of 50 mL of 1 M NH_3_ under stirring conditions. The reaction mixture was stirred until a black-colored colloidal solution was formed. The ferrofluid was separated using a neodymium alloy magnet of 5000 G and was washed using distilled water. Fe_3_O_4_ nanoparticles were stored at 4 °C to prevent oxidation to maghemite [13,14,15].

### 2.3. Synthesis of the NS

NS synthesis was done using a published procedure [16] with slight modifications. The NS were prepared using 1.5 g of β-CD and 0.856 g of diphenylcarbonate (DPC) (molar ratio 1:4). Homogenized anhydrous β-CD and DPC were placed in an Erlenmeyer flask. The mixture was heated to 90 °C under an ultrasound bath and left to react for 6 h. The reaction mixture was left to cool, and the obtained white powder was broken down roughly with an agate mortar. The solid was repeatedly washed with distilled water to remove unreacted β-CD and with ethanol to remove unreacted DPC and phenol, which was a byproduct of the reaction. Afterwards, the solid was subsequently extracted by a Soxhlet apparatus with acetone for 48 h. Finally, the solid was dried at 60 °C in an oven for 48 h and stored at 25 °C for further use.

### 2.4. Decoration of NS with Fe_3_O_4_ Nanoparticles

A determined mass of NS (20 mg) was suspended on 10 mL of magnetite nanoparticles. The suspension was allowed to settle and then centrifuged at 15,000 rpm for 20 min. NS changed from white to black once the magnetite nanoparticles had been deposited on the polymer. NS functionalized with magnetite were dried under vacuum and then exposed to a neodymium alloy magnet to evaluate the magnetic response of the magnetic polymer.

### 2.5. Characterization of NS

Proton nuclear magnetic resonance (^1^H-NMR), X-ray powder diffraction (XRPD), thermogravimetric analysis (TGA), scanning electron microscopy (SEM), Fourier transform infrared spectroscopy (FT-IR), BET analysis, dynamic light scattering (DLS), and transmission electron microscopy (TEM) analyses were performed to confirm the formation of NS. Further details can be found in the Supplementary Information, Table S1, Figures S1–S7.

### 2.6. Characterization of Fe_3_O_4_ Nanoparticles

XRPD, TGA, TEM, Z-potential, dynamic light scattering (DLS), energy dispersive spectroscopy (EDS), saturation magnetization (VSM), and selected area electron diffraction (SAED) analyses were carried out in order to confirm the presence of Fe_3_O_4_ nanoparticles [13,14,15]. Details for the zero field-cooled (ZFC) and field-cooled (FC), DLS, TGA and XRPD analyses can be found in the Supplementary Information, Figures S8–S12.

### 2.7. Characterization of NS/Pesticide Complexes

Determination of the ^1^H-NMR spectra of NS-pesticide complexes was done using a Bruker Avance 400 MHz spectrometer, with 16 scans. Stock solutions of the NS/pesticide complexes were prepared in deuterated DMSO. Deuterated DMSO was chosen as solvent since the NS is not soluble in deuterated water/chloroform, and previous studies have shown that DMSO does not interfere in the formation of the inclusion compounds [17]. XRPD patterns of the NS/pesticide complexes were recorded using a high-power powder Siemens/Bruker D5000 diffractometer equipped with a Cu anode and Ni target filter. The diffractometer had a current of 40 kV/40 mA and a scan speed of 0.05°/s. The samples were analyzed over a 2ϴ angle range of 2–30°.

TGA of NS–P complexes were performed on a TGA-4000 Pyris 6 thermogravimetric analyzer over a temperature range of 25–400 °C. Approximately 10 mg of the sample was placed on a pan, which was then subjected to the abovementioned conditions under a nitrogen atmosphere. The surface morphology of the NS/pesticide complexes was determined using a LEO VP1400 analytical scanning electron microscope (SEM) equipped with an Oxford 7424 energy dispersive spectrometer (EDS). NS/pesticide complexes were centrifuged at 12,000 rpm and then dried on vacuum. Solid samples were sputter coated with gold and prepared by the application of carbon films coated on aluminum stubs.

UV–VIS spectra of the NS/pesticide complexes were recorded using a Perkin Elmer Lambda 25 UV–VIS spectrometer. Measurements were carried out over a range of 250–500 nm using deionized water as a reference.

### 2.8. Characterization of NS Decorated with Fe_3_O_4_ Nanoparticles

Saturation magnetization (VSM), Zero field cooled (ZFC) and field-cooled (FC) of NS decorated with magnetite, both in powder form, was measured using VSM magnetometer at room temperature. The surface morphology of the NS functionalized with magnetite was determined using a LEO VP1400 analytical scanning electron microscope (SEM) equipped with an Oxford 7424 energy dispersive spectrometer (EDS).

### 2.9. Sorption Studies

Sorption studies were carried out using previously reported procedures with slight modifications [9,10]. A range of concentrations of each pesticide (0.1–8 mM) was prepared at pH 9 using a NaHCO_3_ buffer (0.01 M). Alkaline pH conditions were chosen throughout this study to ensure proper solubility for both pesticides. A set amount of NS (20 mg) was added to a fixed volume (10 mL) of pesticide solution in a glass container.

The container was sealed and placed in a magnetic stirrer at room temperature. The pesticide concentration was determined at different times (30, 60, 180, 300, and 360 min) using a spectrophotometer to monitor the absorbance changes at λ_max_.

The equilibrium pesticide concentration in the polymer phase (*Qe*) removed from the solution is defined by Equation (1), where *Co* is the initial pesticide concentration, *Ce* is the final pesticide concentration, *V* is the solution volume, and *m* is the polymer mass:

(1) $$Qe=\frac{\left(Co-Ce\right)\;V}{m},$$

### 2.10. Kinetic Studies

Kinetic adsorption experiments of both pesticides were carried out in the presence of 20 mg of NS in a 10 mL solution of each guest molecule. A constant agitation speed was applied using a magnetic stirrer for specific contact periods (5–300 min). The samples were collected at different time intervals, and the supernatant was analyzed using UV–VIS spectroscopy by monitoring changes at the wavelength of maximum absorbance. To investigate the adsorption mechanism, the non-linear forms of the pseudo-first-order and pseudo-second-order kinetic rate equations are given in Equations (2) and (3), respectively:

(2) $$Qt=Qe\;\left(1-e^{-k1t\;}\right)\;$$

(3) $$Qt=\frac{K2Qe^{2}}{1+k2Qet}\;t\;$$

where *Qt* and *Qe* represent the amounts of pesticide adsorbed at equilibrium (mg/g) and at time “*t*” (min). *K*_1_ (min^−1^) and *K*_2_ (g/mg min) are the pseudo-first-order and pseudo-second-order rate constants, respectively.

### 2.11. Isotherm Studies

A Sips isotherm [18] was used to analyze the experimental equilibrium data. This model is represented by Equation (4):

(4) $$Qe=\frac{Qm\;{\left(KsCe\right)}^{ns}}{1+\;{\left(KsCe\right)}^{ns}}$$

where *Qm* is the Sips adsorption capacity (mg/g), *Ks* represents the affinity constant for a heterogeneous solid (L/mg), and *ns* is the heterogeneity parameter. At low adsorbent concentrations, the Sips isotherm reduces to a Freundlich isotherm, whereas at high adsorbent concentrations, the Sips isotherm is better represented by monolayer adsorption, characteristic of a Langmuir isotherm.

## 3. Results and Discussion

### 3.1. ^1^H-NMR Spectra of the NS-Pesticide Complexes

**Figure 1:**
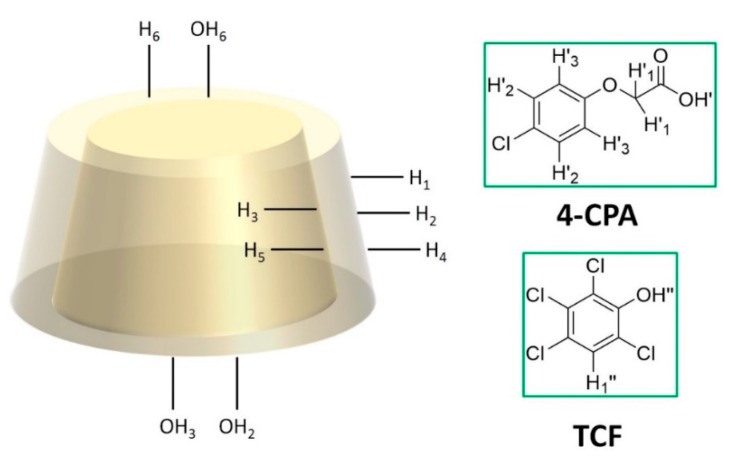
Proton assignment for β-cyclodextrin (β-CD) and both pesticides (4-chlorophenoxyacetic acid (4-CPA) and 2,3,4,6-tetrachlorophenol (TCF)).

The formation of NS–CPA and NS–TCF inclusion compounds was confirmed using ^1^H-NMR spectroscopy. The proton assignment for each pesticide and CD is shown in Figure 1. Proton signals from the guest molecules showed high-field chemical shifts, possibly due to screening effects caused by the spatial restriction of the guests included inside the cavities of the NS [19]. H3 and H5 signals (internal proton signals of the NS host molecule) also showed chemical shifts, confirming the inclusion.

The OH_2_ and OH_3_ chemical shifts can be attributed to changes in the electrostatic field due to the presence of the –Cl functional groups in the pesticides. Chemical shifts for 4-CPA and TCF protons after the formation of the inclusion compound are shown in Table 1 and Table 2, respectively. The ^1^H-NMR spectra for the NS–4-CPA and NS–TCF complexes can be found in Supplementary Material, Figure S7.

### 3.2. XRPD of the NS–Pesticide Complexes

**Figure 2:**
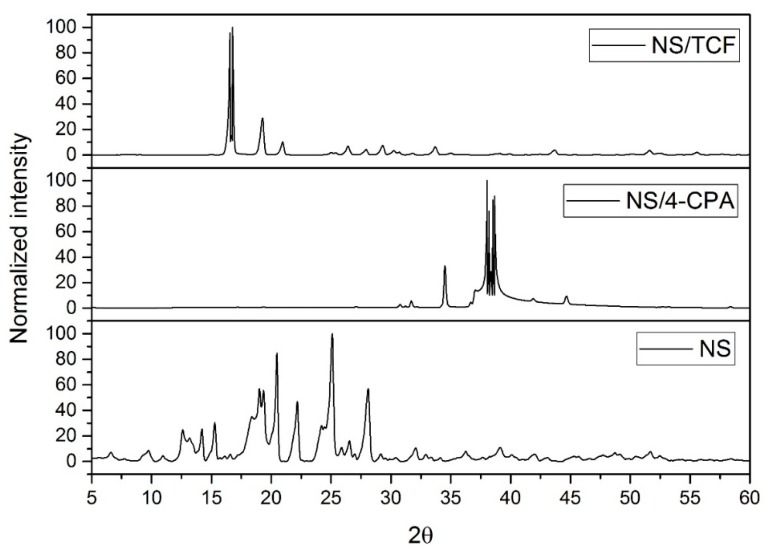
X-ray powder diffraction (XRPD) analysis for nanosponges (NS) and NS–pesticide complexes.

Complex formation was also confirmed by XRPD analysis. Figure 2 shows the XRPD patterns of the pesticide-loaded NS. In both diffraction patterns, the characteristic peaks of the pesticide were reduced [20,21,22,23,24,25,26,27]. It is also possible to confirm the lack of native NS and β-CD in the inclusion compound.

### 3.3. TGA of the NS–Pesticide Complexes

**Figure 3:**
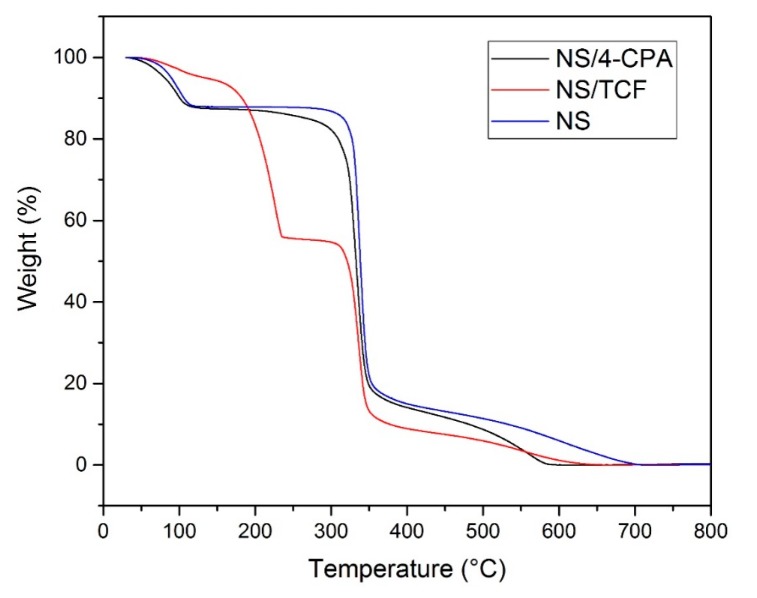
Thermogravimetric analysis (TGA) analysis for nanosponges (NS), the NS–2,3,4,6-tetrachlorophenol (TCF) complex and the NS–4-chlorophenoxyacetic acid (4-CPA) complex.

Figure 3 shows the TGA analysis of pesticide-loaded NS. Weight loss occurred at 30–100 °C due to the loss of water molecules from the nanosponge. The NS–pesticide complexes started to degrade at 100 °C, followed by the main degradation step at over 300 °C. The NS–CPA thermogram is similar to that of plain NS, as CPA started to evaporate/decompose at 315 °C. The NS–TCF thermogram shows weight loss at 185 °C, corresponding to the degradation of TCF. These results are in agreement with the literature, suggesting that encapsulation improves the thermal stability of the guests [20,21,22,23,24,25,26,27].

### 3.4. SEM and EDS Analyses of the NS Complexes

**Figure 4:**
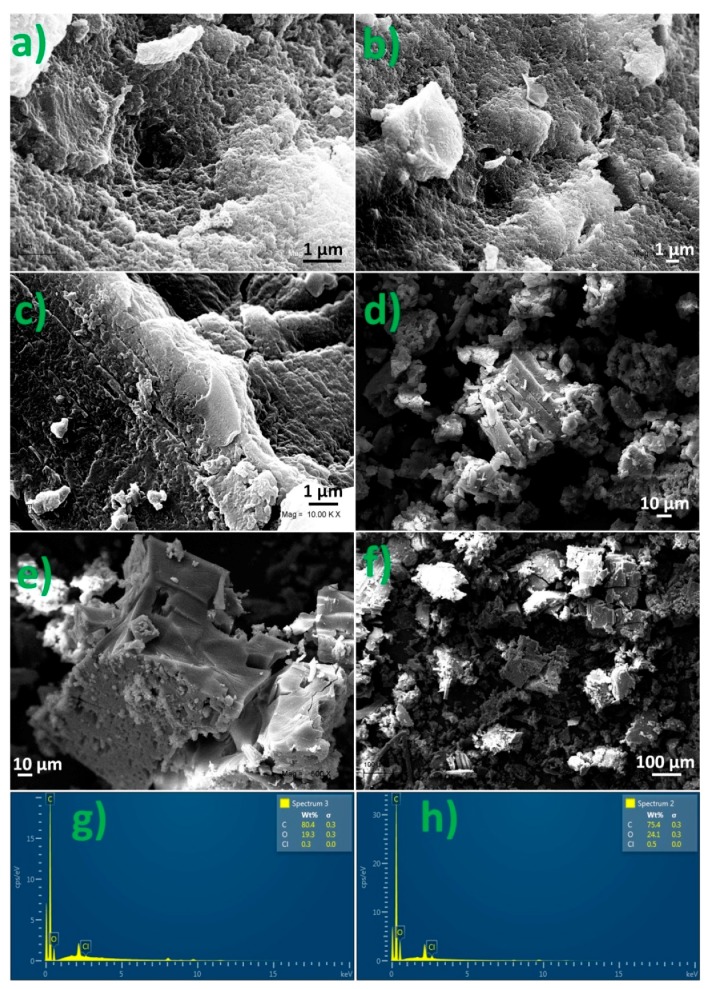
SEM image of nanosponges (NS) (**a**,**b**), NS–2,3,4,6-tetrachlorophenol (TCF) (**c**,**d**), and NS–4-chlorophenoxyacetic acid (4-CPA) (**e**,**f**), and energy dispersive X-ray spectroscopy (EDS) analysis of the NS–pesticide complex (**g**,**h**).

The formation of the NS–pesticide complexes was also confirmed by SEM and energy dispersive X-ray spectroscopy (EDS) analyses. Changes in the surface morphology were observed, as the pores of the NS were partially filled with host molecules, as seen in Figure 4.

EDS provides the elemental composition and weight percentage of the inclusion compound. The graph shows the weight percentage and the presence of chlorine, oxygen, and carbon.

### 3.5. Sorption Studies

#### 3.5.1. Calibration Curves and Determination of Molar Attenuation

The molar attenuation of both guests (ε-CPA and ε-TCF) was determined using the Beer–Lambert equation. The absorbance for a set of pesticide solutions was recorded using UV–VIS spectroscopy (230 nm). The molar attenuation for both pesticides is shown in Table 3, Figure S13.

#### 3.5.2. Sorption Efficiency Comparison

**Figure 5:**
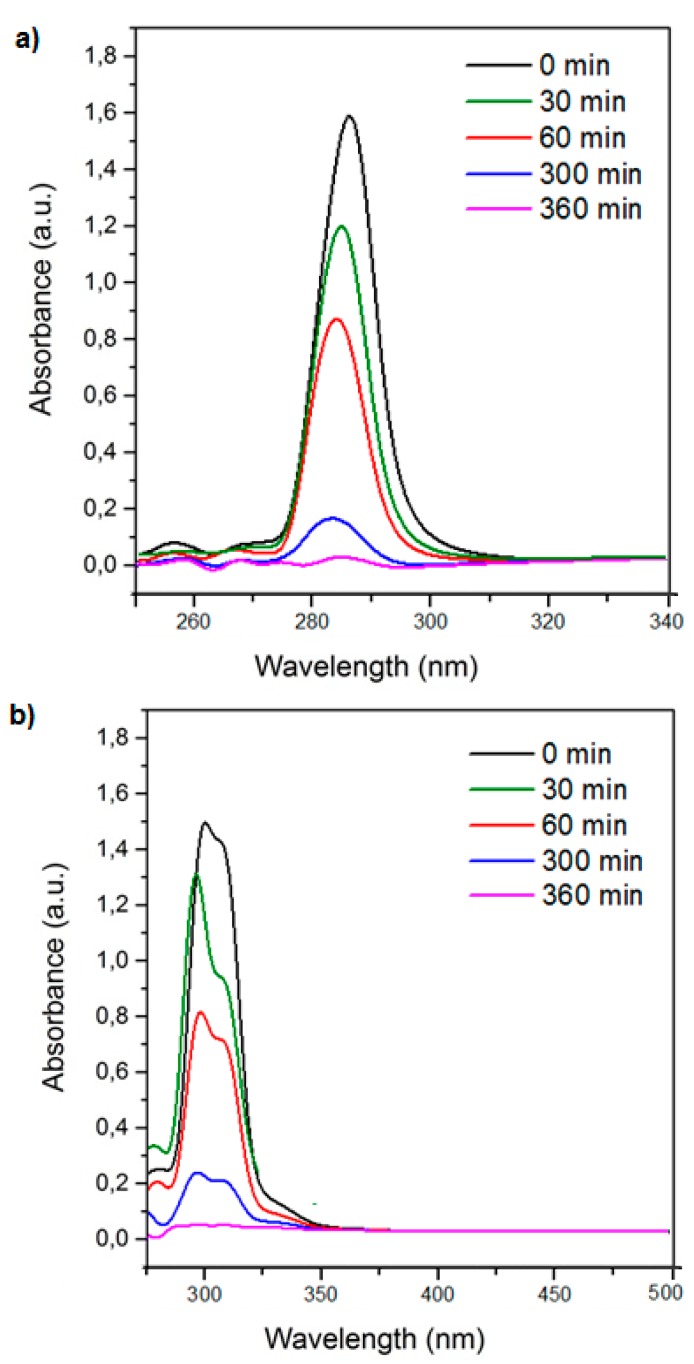
UV–VIS spectra for 4-chlorophenoxyacetic acid (4-CPA) (**a**) and 2,3,4,6-tetrachlorophenol (TCF) (**b**) at different contact times.

Figure 5 shows the UV–VIS spectra of both pesticides at different contact times. The maximum absorbance for each guest molecule decreased as the contact time increased.

Ce was determined using the calibration curves obtained from previous Section 3.5.1, and *Qe* was determined using Equation (1).

**Figure 6:**
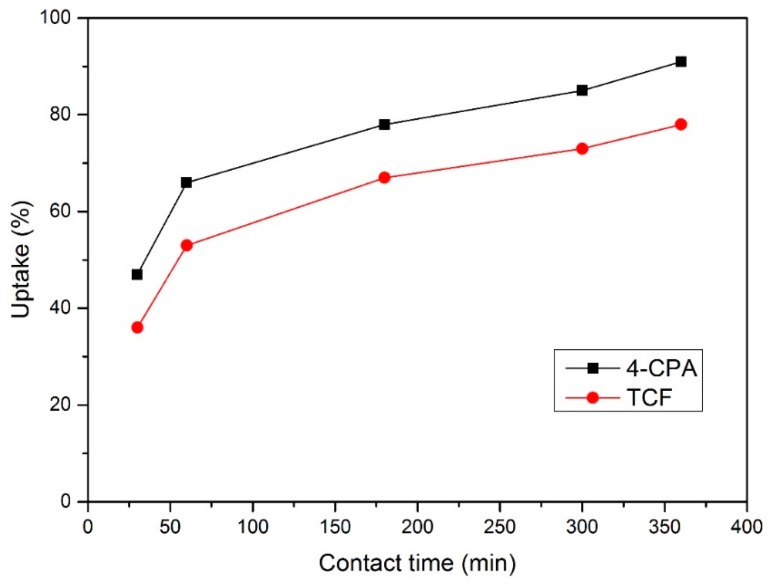
Uptake percentage for each pesticide.

The *Qe* and *Ce* values are shown in Table 4 and Figure 6. For all experiments, the solution volume was 7 mL, the pesticide concentration was 0.01 mM, and the amount of NS used was 20 mg.

According to these results, NS are indeed viable materials for the removal of pesticides from aqueous solution due to the high adsorption of chlorinated organic compounds and because a contact time of 120 min was enough to reduce the guest concentration to the µM level. Table 5 compares the *Ce* and *Qe* values obtained for both guest molecules using different sorbents: NS and GAC. The pesticide concentration was 0.01 mM, and the contact time was 360 min.

The sorption capacities of the pesticide adsorbates were as follows: NS > GAC. Differences in the sorption capacity can be explained by the surface areas of the sorbents. The NS have lower surface areas (3 m^2^/g) than GAC (600 m^2^/g).

However, the sorption capacity of NS is higher than that of GAC. Potentially, guest molecules are not only adsorbed by the surface of NS, but also transported to the nanoporous bulk polymer during the inclusion process. NS also undergo swelling, which increases their surface area when they are in a hydrated state [28].

Swelling might occur due to water molecules entering the hydrophilic NS pores and the aggregation of some of the polymer sites through non-covalent interactions, resulting in an interconnected network.

On the other hand, materials such as GAC are rigid and do not show large changes in their surface area while in a hydrated state [28].

### 3.6. Kinetic Studies

**Figure 7:**
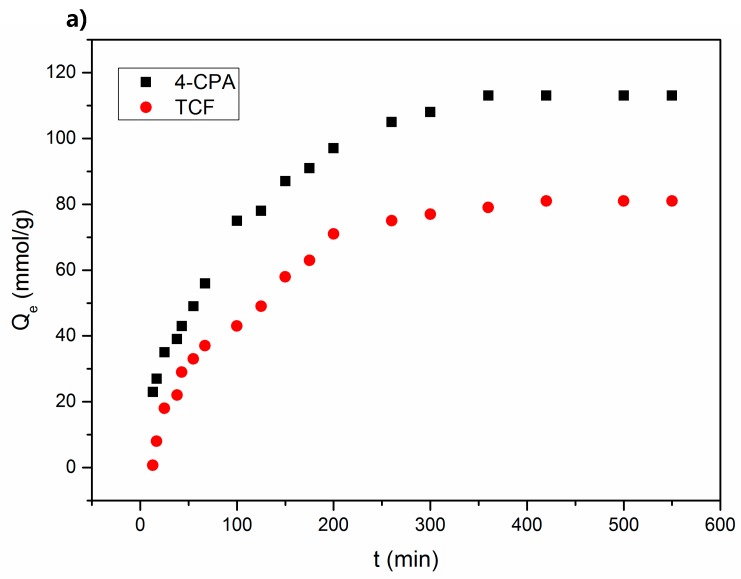

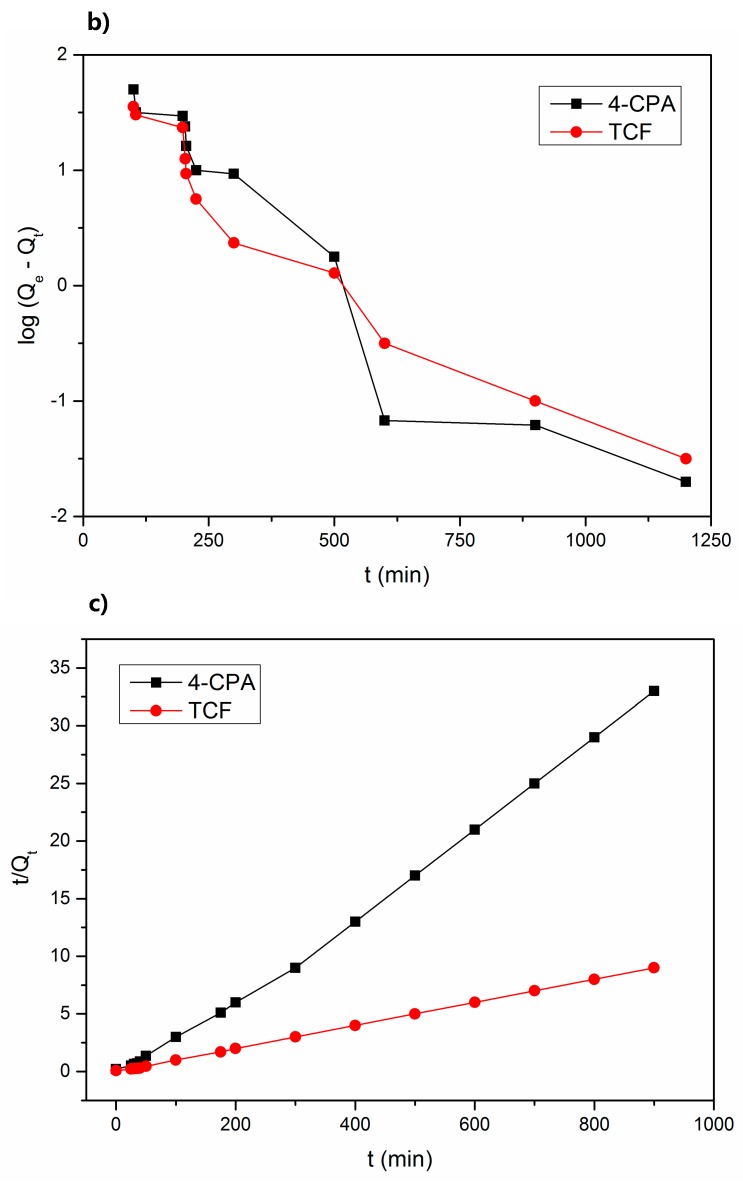
Plots of the sorption kinetics (**a**), pseudo-first order kinetics (**b**), and pseudo-second order kinetics (**c**) for both pesticides.

Figure 7 shows the effect of contact time on the sorption capacities of the NS and linear plots for the pseudo-first-order and pseudo-second-order models. The sorption capacity increased rapidly during the initial stages of contact and then increased more gradually until equilibrium was reached. The time taken to reach equilibrium during the formation of the NS–TCF and NS–CPA complexes (both 150 mg/L) was 48 h.

As seen from Table 6 and the obtained correlation coefficients, pesticide sorption by the NS was better described by the pseudo-second-order model, which relates specifically to chemisorption [29].

The values of both *K*_1_ and *K*_2_ were higher for CPA than for TCF. The NS may show better sorption capacity and greater kinetics towards the former guest, such that the formation of the NS–CPA complex is more favored than the formation of the NS–TCF complex.

### 3.7. Sorption Isotherms

**Figure 8:**
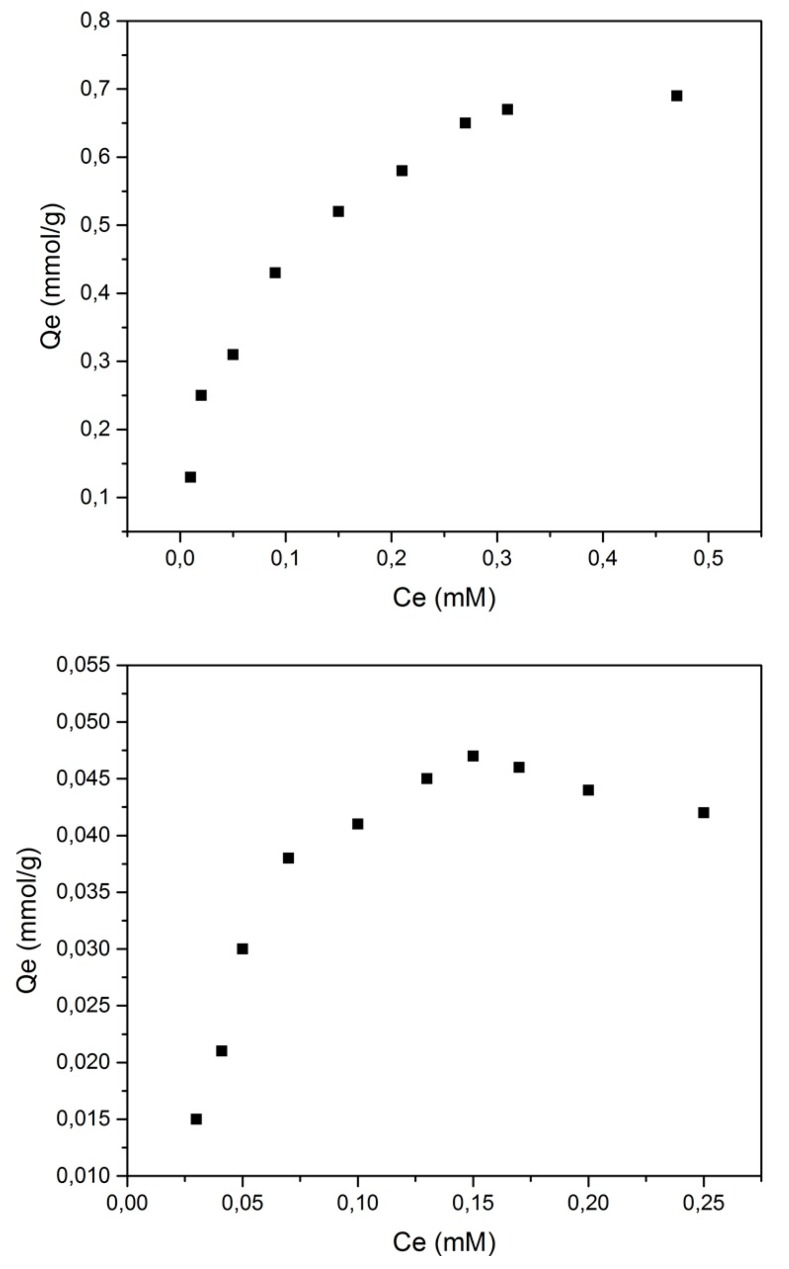
Sorption isotherms for 4-chlorophenoxyacetic acid (4-CPA) (**upper**) and 2,3,4,6-tetrachlorophenol (TCF) (**lower**).

Figure 8 shows the sorption behavior of both pesticides (0.01–7 mM) in aqueous solution. The solid lines represent the best fit according to the Sips isotherm [18].

In both cases, *Qe* and *Ce* increased simultaneously. The sorption capacities (*Qm*) were determined from these results, as seen in Equation (4), and are shown in Table 7.

Under these experimental conditions (alkaline pH), TCF exists in its phenolate anion form, whereas CPA exists as a carboxylate anion. The NS reached saturation at low *Ce* levels (5 mM) with CPA and at even lower *Ce* levels (0.15 mM) with TCF. TCF is a larger adsorbate than CPA and, thus, is expected to show attenuated sorption due to steric effects and reduced inclusion site accessibility [20,21,22].

The difference in electron density for both guests may also contribute to the varying binding affinity for the NS polymer, as the TCF anion charge is delocalized on the aromatic ring, whereas the CPA anion charge resides on the carboxylate.

### 3.8. Reutilization of the NS

**Figure 9:**
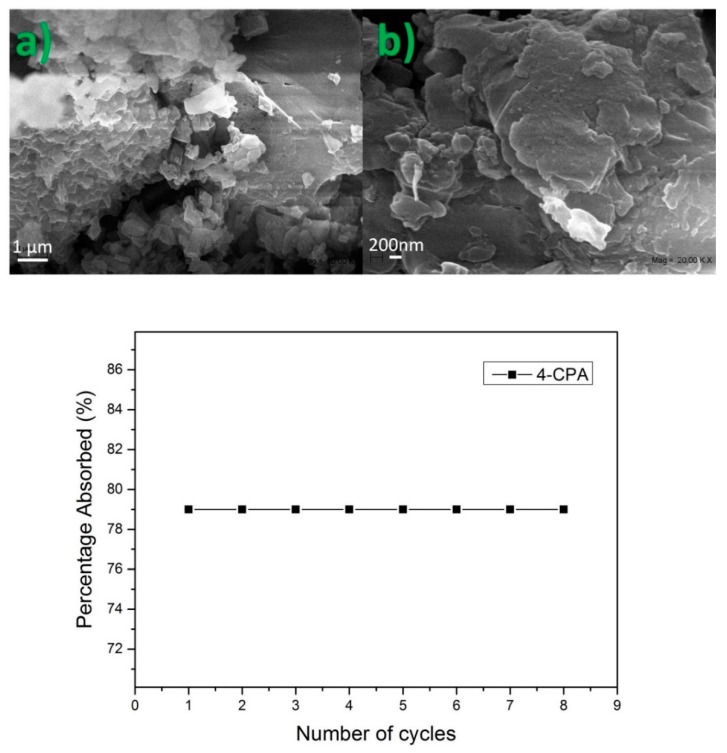
SEM images of nanosponges (NS) after a repeated number of cycles (**a**,**b**). Percentage of 4-chlorophenoxyacetic acid (4-CPA) adsorbed after a repeated number of cycles.

To make the NS cost effective, it should be possible for the polymers to be reutilized several times. Reutilization of the NS was studied by repetitive adsorption experiments using the same polymer. The NS were regenerated by washing with ethanol or ethyl acetate and milli-Q water. Figure 9 shows that NS can maintain their effectiveness after many cycles. SEM images showed prominent changes in the surface of NS after the repeated cycles as the pores were filled with the guests.

### 3.9. NS Decorated with Magnetite NPs

#### 3.9.1. Characterization of Fe_3_O_4_ Nanoparticles

**Figure 10:**
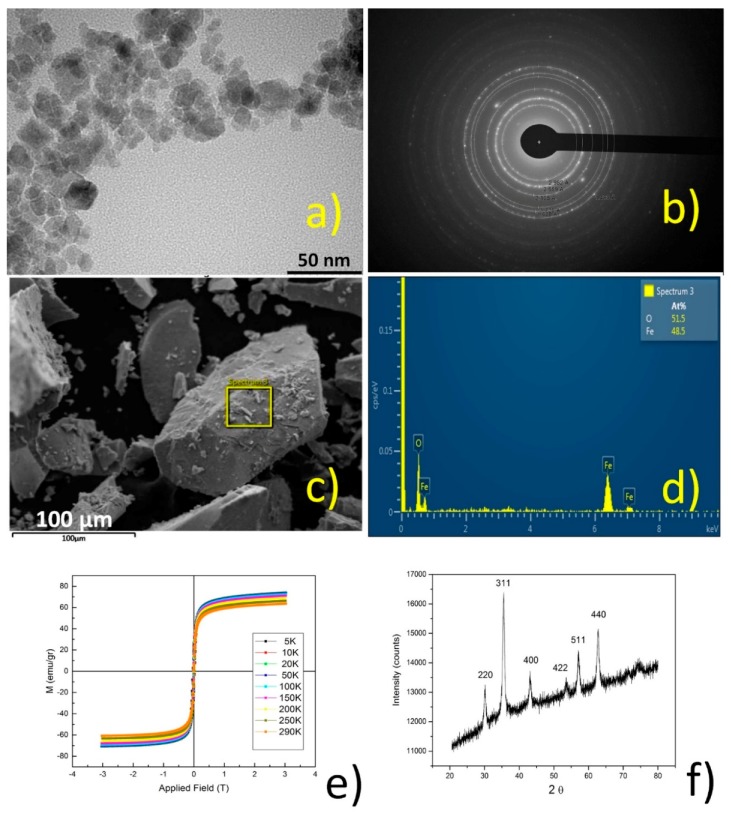
TEM (**a**); selected area electron diffraction (SAED) (**b**); SEM (**c**); EDS (**d**); magnetization saturation (VSM) (**e**); and XRPD (**f**) analysis for Fe_3_O_4_ nanoparticles.

Figure 10 shows TEM, SAED, SEM, EDS, VSM, and XRPD analyses for magnetite nanoparticles. A TEM micrograph of magnetite nanoparticles shows structures with an average particle diameter of around 10 nm. EDS provided the elemental compositions and weight percentages of iron and oxygen. It is evident that both Fe and O were present on the synthetized nanomaterials, with weight percentages of 51.5% for oxygen and 48.5% for iron. The SAED pattern showed that the synthetized materials were magnetite nanoparticles, as all diffraction rings corresponded to lattice planes in the Fe_3_O_4_ crystal structure. The XRPD measurement of magnetite nanoparticles was analyzed. The obtained data shown in Figure 10 were compared with the standard data of bare magnetite. Peaks for uncoated magnetite were obtained at 2ϴ = 30.25, 35.75, 43.35, and 57.35 with the following indices: (220), (311), (400), (422), (511), and (440), Figures S11-12. Through comparison with the standard data, shown in Figure S12, it was possible to confirm the formation of magnetite nanoparticles. The VSM of bare magnetite shows that the value of saturation magnetization is 63 emu/g. The graph also shows that remanence and coercivity values are close to zero, so it is possible to confirm the superparamagnetic nature of the magnetite nanoparticles [13,14,15].

#### 3.9.2. Characterization of NS Decorated with Fe_3_O_4_ Nanoparticles

**Figure 11:**
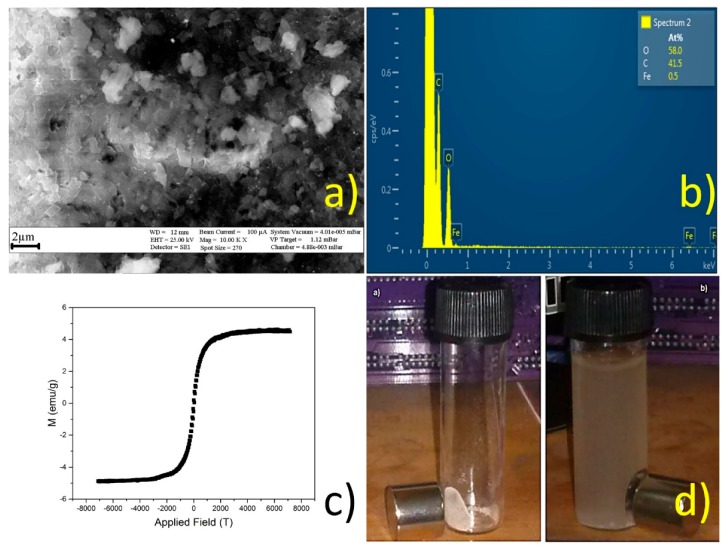
SEM (**a**); EDS (**b**); VSM (**c**); and magnetic response of nanosponges (NS) decorated with magnetite nanoparticles (**d**).

Figure 11 shows the SEM, EDS, and VSM analyses, and the magnetic response for NS functionalized with magnetite nanoparticles.

Magnetite nanoparticles were successfully deposited on the NS and retained their superparamagnetic properties after being immobilized on the polymer [13,14,15].

**Figure 12:**
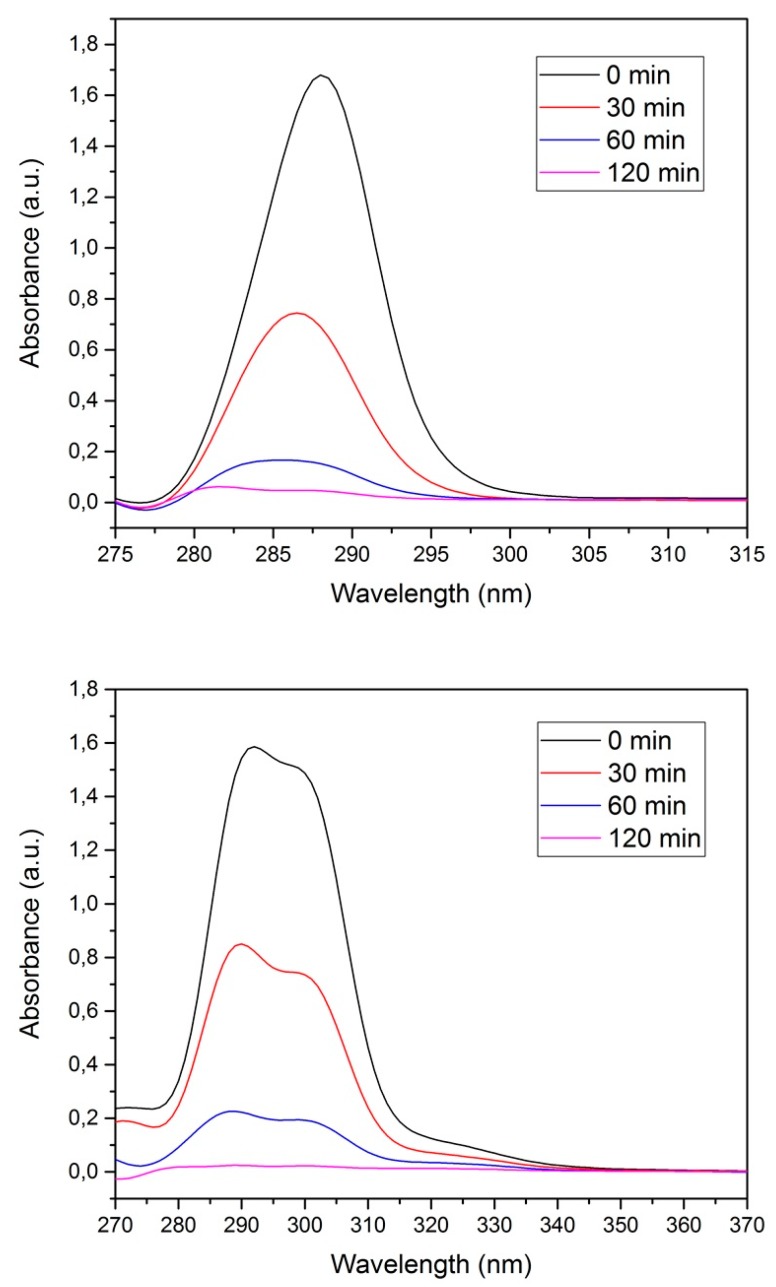
UV–VIS spectra for 4-chlorophenoxyacetic acid (4-CPA) (**upper**) and 2,3,4,6-tetrachlorophenol (TCF) (**lower**) after contact with NS decorated with magnetite.

NS decorated with magnetic NPs were evaluated on their ability to remove aromatic chlorinated compounds from aqueous solution, as well as their reusability by retrieving the magnetic polymer using a neodymium magnet. A sample of 20 mg was added to 10 mL of a pesticide solution, followed by incubation for one hour. The magnetic NS were separated from the solution phase with the neodymium magnet, and the aqueous solution was collected in order to determine the residual concentration of the pesticide using UV–VIS [9,10]. The UV spectra of the pesticide showed that NS retain, and might even improve, their efficiency after being decorated with magnetite nanoparticles, as the magnetic polymer outperforms native NS within a couple of hours. Those results are summarized in Figure 12 and Table 8.

### 3.10. Loading Capacity

**Figure 13:**
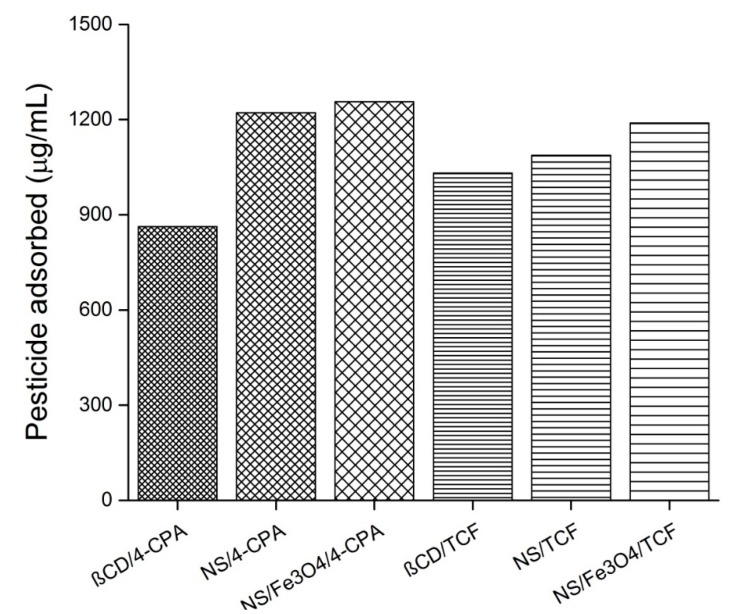
Amount of pesticide adsorbed with β-Cyclodextrin (β-CD), nanosponges (NS), and Fe_3_O_4_/NS. Other abbreviations: 4-chlorophenoxyacetic acid (4-CPA), and 2,3,4,6-tetrachlorophenol (TCF).

Figure 13 shows the loading capacities of β-CD, NS, and NS decorated with Fe_3_O_4_ nanoparticles as follows: NS/Fe_3_O_4_ > NS > β-CD. These results are in agreement with those obtained in the previous section, as NS showed improved efficiency after being decorated with Fe_3_O_4_ nanoparticles, in comparison to native β-CD and NS [9,10].

## 4. Conclusions

In this work, we successfully synthesized and characterized cyclodextrin NS for the removal of pesticides from aqueous solutions. β-CD-based NS is able to efficiently form a complex with both CPA and TCF. Sorption studies showed that NS have favorable sorption for chlorinated aromatic guests in solution. Overall, the sorption capacity and binding affinity of the sorbents were shown to be greater for NS, despite GAC having a greater surface area. Complexes formed with TCF were shown to be weaker than those formed with CPA due to the size of the adsorbate and the steric effects in the hydroxyl region that arise from the NS network. SEM, EDS, and VSM analyses also showed that NS could be a great substrate to stabilize nanoparticles, thus giving the polymer useful and additional properties, as decorated NS outperformed native NS in terms of pesticide removal efficiency and was also easily recovered from the solution by the use of external magnetic fields. NS materials may eventually be considered to be an improved technology for pollutant removal from soil and aquatic environments, as they are efficient, low cost, and reusable.

## Figures and Tables

**Table 1:** ^1^H-NMR assignments and chemical shifts for pesticides and the nanosponge (NS)—4-chlorophenoxyacetic acid (4-CPA) complex.

**H 4-CPA**	**δ 4-CPA (ppm)**	**δ NS–4-CPA (ppm)**	**Δδ (ppm)**
OH’	13.035	12.926	−0.106
H’ 2	7.322	7.319	−0.002
H’ 3	6.937	6.934	−0.004
H’ 4	4.682	4.677	−0.004
**H NS**	**δ NS (ppm)**	**δ NS–4-CPA (ppm)**	**Δδ (ppm)**
OH 2	5.704	5.716	0.012
OH 3	5.670	5.670	0.001
OH 6	4.440	4.447	0.006
H 1	4.827	4.829	0.002
H 3	3.627	3.636	0.009
H 5	3.572	3.636	0.003
H 6	3.572	3.574	0.002

**Table 2:** ^1^H-NMR assignments and chemical shifts for pesticides and NS–2,3,4,6-tetrachlorophenol (TCF) complex.

**H TCF**	**δ TCF (ppm)**	**δ NS–TCF (ppm)**	**Δδ (ppm)**
H 1	7.215	7.136	−0.079
H 2	5.948	5.678	−0.270
**H NS**	**δ NS (ppm)**	**δ NS–TCF (ppm)**	**Δδ (ppm)**
OH 2	5.704	5.704	0.000
OH 3	5.670	5.667	0.003
OH 6	4.440	4.441	−0.001
H 1	4.827	4.825	0.002
H 3	3.627	3.625	0.002
H 5	3.572	3.570	0.002
H 6	3.655	3.653	0.002

**Table 3:** Molar attenuation for 4-chlorophenoxyacetic acid (4-CPA) and 2,3,4,6-tetrachlorophenol (TCF).

Pesticide	ε (mM/cm)
4-CPA	10.10
TCF	8.27

**Table 4:** Ce and Qe values for both pesticides and at different contact times.

Pesticide	Contact Time (Min)	*C_e_* (mM)	*Q_e_* (mmol/g)	Uptake
4-CPA	30	5.33 × 10^−3^	1.63 × 10^−3^	47%
4-CPA	60	3.41 × 10^−3^	2.33 × 10^−3^	66%
4-CPA	180	2.21 × 10^−3^	2.73 × 10^−3^	78%
4-CPA	300	1.53 × 10^−3^	2.95 × 10^−3^	85%
4-CPA	360	9.03 × 10^−4^	3.19 × 10^−3^	91%
TCF	30	6.41 × 10^−3^	1.27 × 10^−4^	36%
TCF	60	4.67 × 10^−3^	1.87 × 10^−3^	53%
TCF	180	3.35 × 10^−3^	2.33 × 10^−3^	67%
TCF	300	2.70 × 10^−3^	2.57 × 10^−3^	73%
TCF	360	2.21 × 10^−3^	2.73 × 10^−3^	78%

**Table 5:** Ce and Qe values obtained for two sorbents: nanosponges (NS) and granular activated carbon (GAC).

Complex	*Ce* (mM)	*Qe* (mmol/g)	Uptake
GAC–4-CPA	2.67 × 10^−3^	2.55 × 10^−3^	73%
GAC–TCF	3.35 × 10^−3^	2.33 × 10^−3^	67%
NS–4-CPA	9.03 × 10^−4^	3.19 × 10^−3^	91%
NS–TCF	2.21 × 10^−3^	2.73 × 10^−3^	78%

**Table 6:** Values obtained for K1 and K2.

Guest Molecule	Concentration (mg/L)	*K*_1_ (min^−1^)	*K*_2_ (mg/g min)	R^2^ (*K*_1_)	R^2^ (*K*_2_)
4-CPA	150	4.45 × 10^−2^	3.66 × 10^−3^	0.967	0.996
TCF	150	3.55 × 10^−2^	1.57 × 10^−3^	0.953	0.993

**Table 7:** *Qm* values for both pesticides.

Guest Molecule	Isotherm	*ns*	*Qm* (mmol/g)	r^2^
4-CPA	Sips	0.78	0.671	0.965
TCF	Sips	0.81	0.077	0.966

**Table 8:** *Ce* and *Qe* values for both pesticides and at different contact times with Fe_3_O_4_/NS.

Pesticide	Contact Time (Min)	*C_e_* (mM)	*Q_e_* (mmol/g)	Uptake
4-CPA	30	4.75 × 10^−3^	1.85 × 10^−3^	53%
4-CPA	60	2.81 × 10^−3^	2.53 × 10^−3^	71%
4-CPA	120	8.89 × 10^−4^	3.19 × 10^−3^	90%
TCF	30	5.98 × 10^−3^	1.41 × 10^−3^	40%
TCF	60	3.97 × 10^−3^	2,11 × 10^−3^	61%
TCF	120	2.13 × 10^−3^	2.75 × 10^−3^	79%

## Supplementary Materials

The following are available online at http://www.mdpi.com/2073-4360/10/9/1038/s1; Figure S1: ^1^H-NMR spectra (400 MHz, DMSO-*d*^6^) of NS and β-CD; Figure S2: XRPD of NS and CD; Figure S3: FT-IR of CD and NS; Figure S4: Nitrogen sorption-desorption isotherm for NS; Figure S5: TEM image of NS; Figure S6: DLS analysis for NS; Figure S7: ^1^H-NMR spectra of NS, NS/4-CPA and NS/TCF (400 MHz, DMSO-*d*^6^); Figure S8: ZFC/FC for magnetite NPs; Figure S9: DLS and Z-potential for magnetite NPs; Figure S10: TGA analysis for magnetite nanoparticles; Figure S11: Response of magnetite nanoparticles to an external magnetic field; Figure S12: Comparison of magnetite XRPD analysis with standard data; Figure S13: Calibration curves for 4-CPA and TCF; Table S1: ^1^H-NMR assignments and chemical shifts for β-CD and NS.
