# Supplementary material for: Removal of Aromatic Chlorinated Pesticides from Aqueous Solution Using β-Cyclodextrin Polymers Decorated with Fe3O4 Nanoparticles
Source: Polymers (Basel). 2018 Sep 19;10(9):1038. doi: 10.3390/polym10091038 (PMC6403796; doi:10.3390/polym10091038)
Supplement: Supplementary file 1 [file polymers-10-01038-s001.zip › polymers-338433-SI.pdf]

Supplementary materials:

Removal of Aromatic Chlorinated Pesticides from Aqueous Solution Using β-Cyclodextrin Polymers Decorated with Fe_3O_4 Nanoparticles.

Sebastián Salazar, Daniel Guerra *, Nicolás Yutronic and Paul Jara *

Department of Chemistry, Facultad de Ciencias, Universidad de Chile, Las Palmeras 3425, Ñuñoa, Santiago 7800024, Chile; sebasalazar@ug.uchile.cl (S.S.); nyutroni@uchile.cl (N.Y.)

* Correspondence: danielguerradiaz@gmail.com (D.G.); pjara@uchile.cl (P.J.); Tel.: +56-988-937-209 (D.G.); +56-229-787-396 (P.J.); Fax: +56-2271-3888 (P.J.)

1. Characterization of β-cyclodextrin (β-CD) polymers (nanosponges)

^{1}H-NMR spectrum of β-CD and nanosponges (NS) is shown on figure S1. Proton signals of NS show chemical shifts in comparison to β-CD spectra, possibly due to the presence of the carbonyl group. This brings us to the conclusion that the reaction between the β-CD monomer and the linkers has occurred [19].

Table S1. ^{1}H-NMR assignments and chemical shifts for β-CD and NS.

	δ βCD (ppm)	δ NS (ppm)	Δδ (ppm)
OH-2	5,721	5,704	-0,017
OH-3	5,670	5,670	0,000
H 1	4,823	4,827	0,004
OH-6	4,452	4,440	-0,011
H 6	3,652	3,655	0,004
H 3	3,618	3,627	0,009
H 5	3,556	3,572	0,016

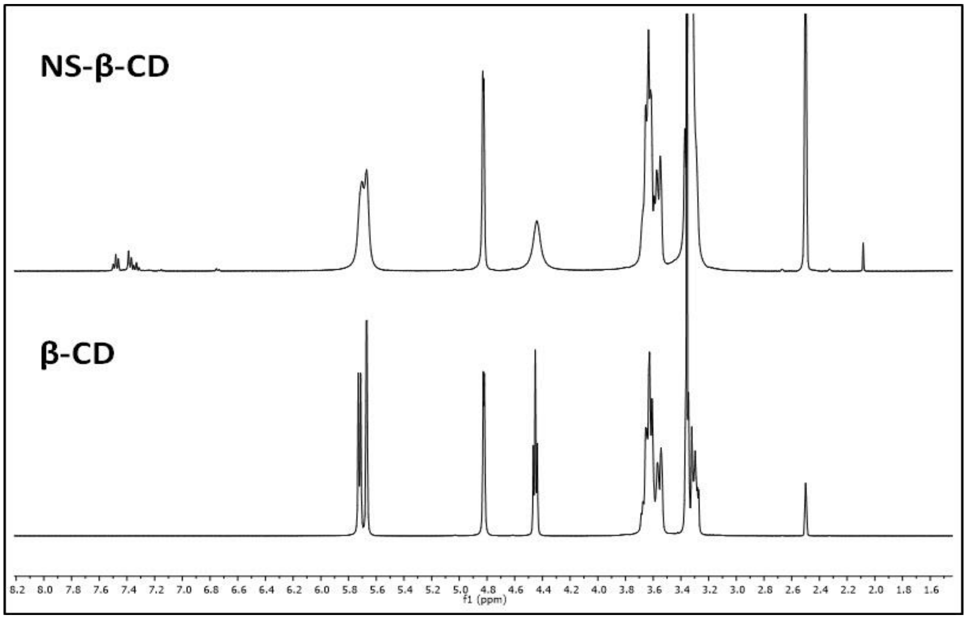

Figure S1. ^{1}**H-NMR spectra (400 MHz, DMSO-*d*^{6}) of NS and β-CD.**

XRPD diffraction patterns (figure S2) show that CD and NS have significant differences as there is a reduction on the number and intensity of peaks by comparison [21, 22].

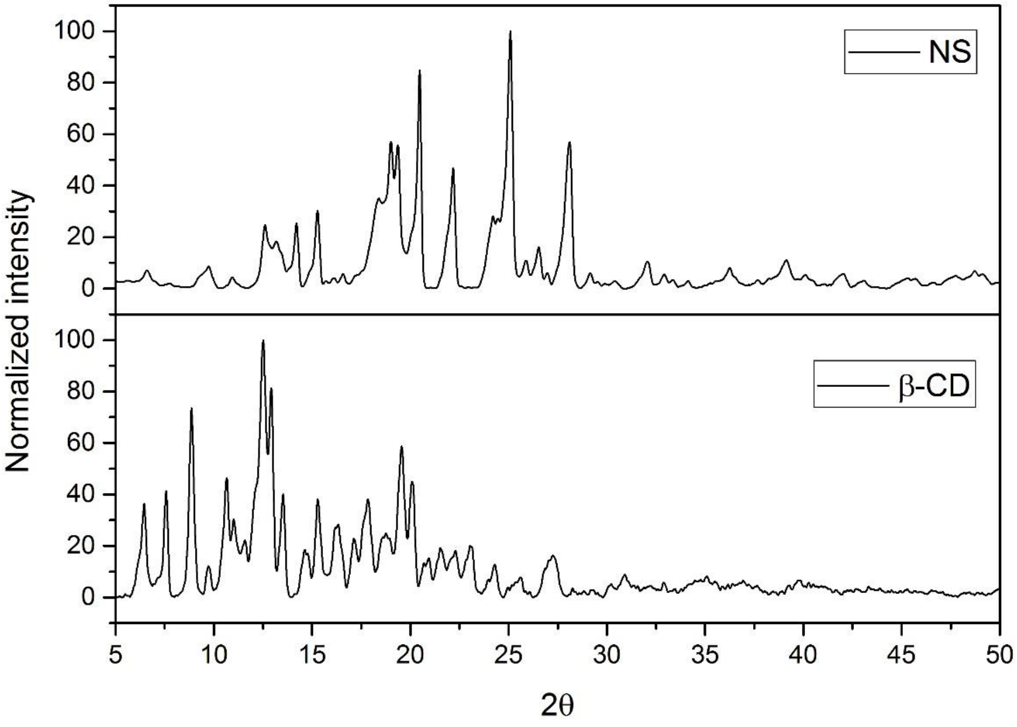

Figure S2. XRPD of NS and β-CD.

FT-IR spectrum of a dried sample of NS was determined using a Spectrum 100 (Perkin Elmer) spectrometer at room temperature in the wavenumber range of 600-4000 cm^{-1} on KBr pellets. FT-IR spectra of NS (figure S3) showed a characteristic peak of carbonyl group at around 1700 cm^{-1} which confirms the formation of the cyclodextrin polymer [20-22]. The carbonyl group peak at around 1700 cm^{-1} is missing on the FT-IR spectra of β-CD which also confirms the formation of the NS.

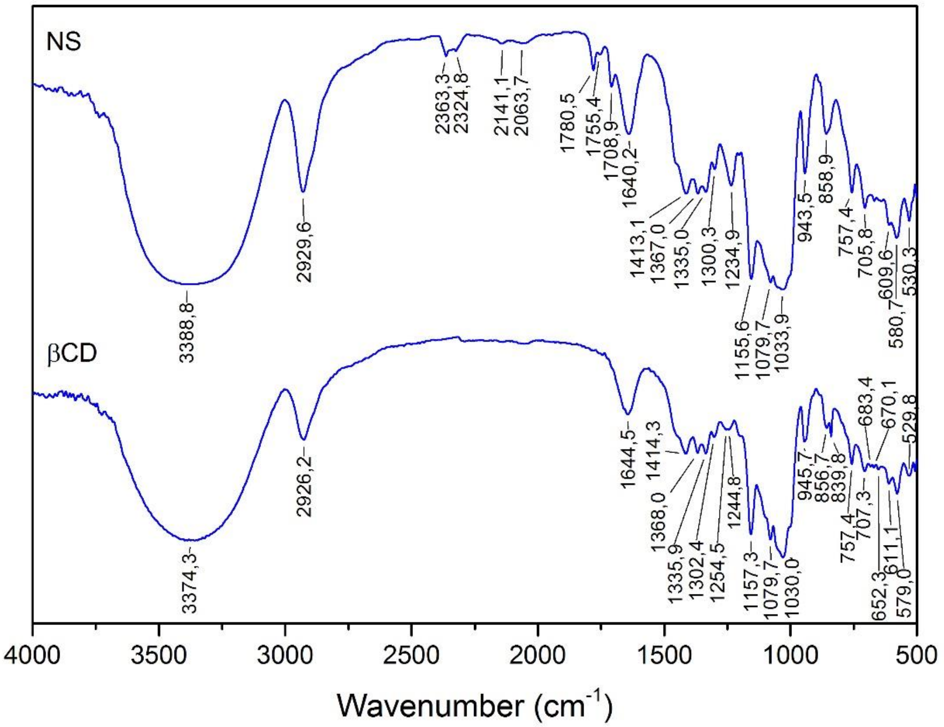

Figure S3. FT-IR spectra of β-CD and NS

BET analysis was performed using a Quantachrome Novawin 11.04 N_2 physisorption machine. Samples were heated at 150°C for a couple of hours to eliminate potential impurities. Nitrogen adsorption-desorption isotherm was measured at 77,350 K. BET results (figure S4) show that NS have a relatively low surface area (3,029 m^2/g) and pore radius of 17 Å. Such result is in agreement with previous studies [20-22] that report that NS can have surface areas as large as 50 m^2/g to less than 1 m^2/g.

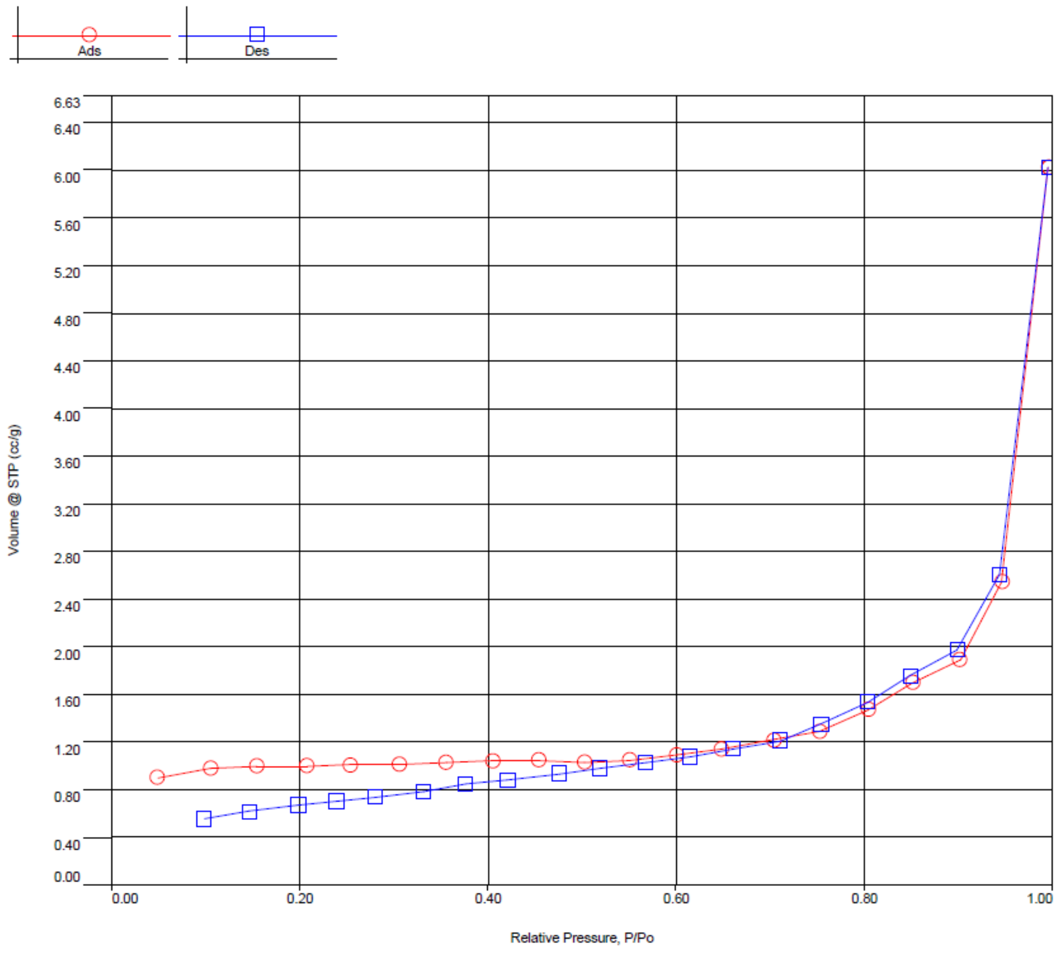

Figure S4. Nitrogen sorption-desorption isotherm for NS. Surface area of 3,029 m^2/g

TEM analysis of NS and Magnetite NPs was performed using a HR-TEM Tecnai ST F20, 800-200 kV instrument. All samples were prepared by dispersing 1 mg in 1 mL of DMSO. Then, 10 μL of the solution was deposited on a copper grid with a film of Formvar. The grid was dried afterwards. TEM images of NS (figure S5) show that NS obtained are of spherical shape and revealed an average size of over 100 nm, which is similar to those obtained in previous studies [20-22].

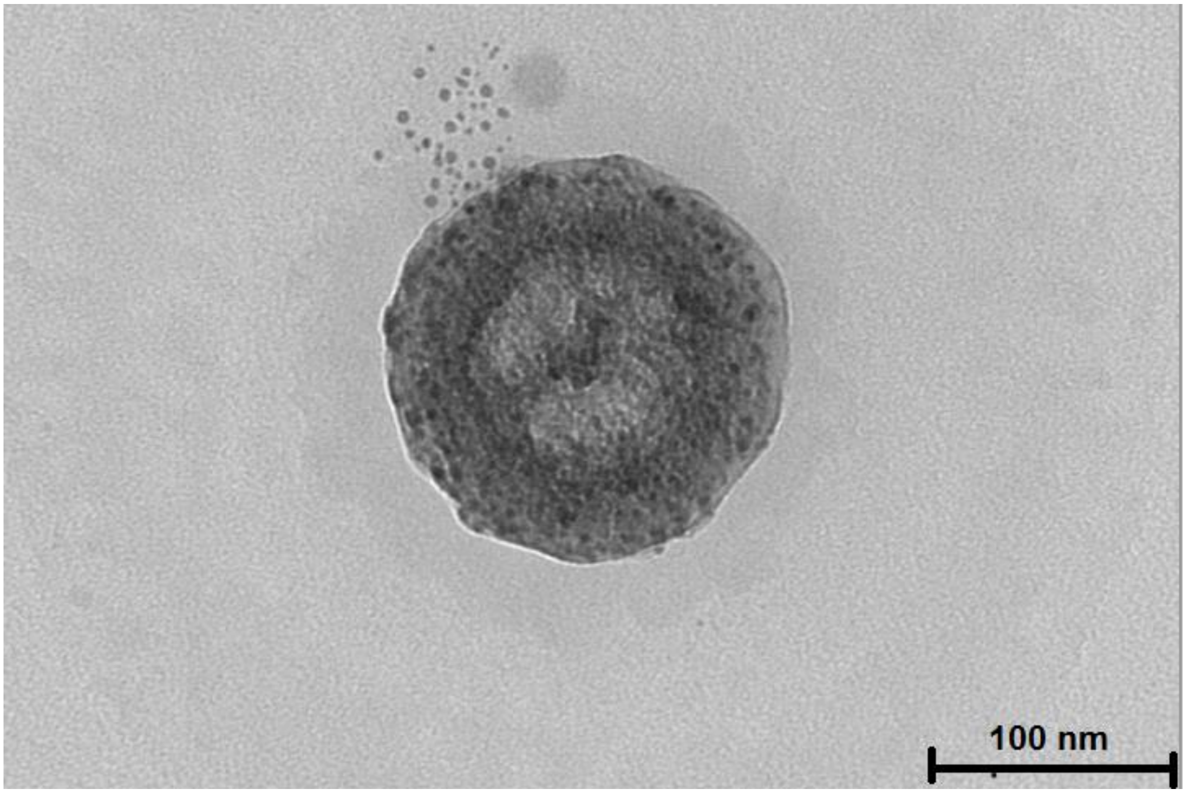

Figure S5. TEM image of NS

Hydrodynamic radius along with Z potential was calculated using DLS Zetasizer NanoS series, Malvern. Hydrodynamic radius and polydispersity index for NS are shown on figure S6. DLS analysis showed a hydrodynamic size for the NS sample of 277 nm with a PDI of 0,575.

**Temperature (°C):**	25,0	**Duration Used (s):**	70
**Count Rate (kcps):**	154,9	**Measurement Position (mm):**	5,50
**Cell Description:**	Clear disposable zeta cell	**Attenuator:**	6

			**Size (d.nm):**	**% Intensity:**	**St Dev (d.nm):**
**Z-Average (d.nm):**	277,5	**Peak 1:**	298,1	91,8	161,4
**PdI:**	0,575	**Peak 2:**	4905	8,2	678,2
**Intercept:**	0,943	**Peak 3:**	0,000	0,0	0,000
**Result quality :**	Good				

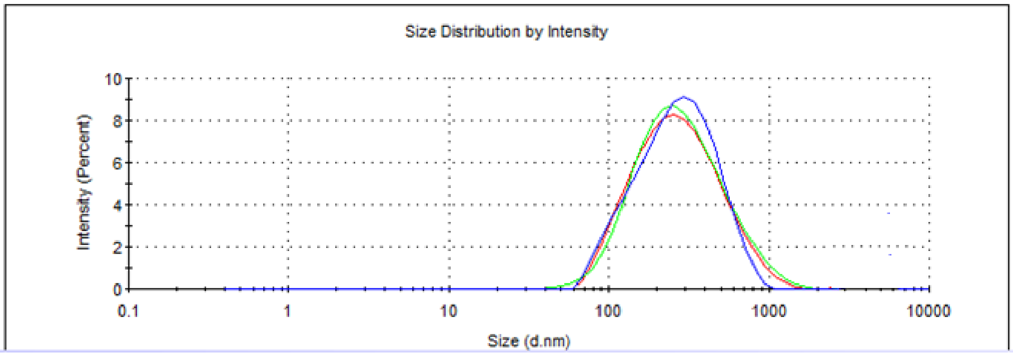

Figure S6. DLS analysis for NS

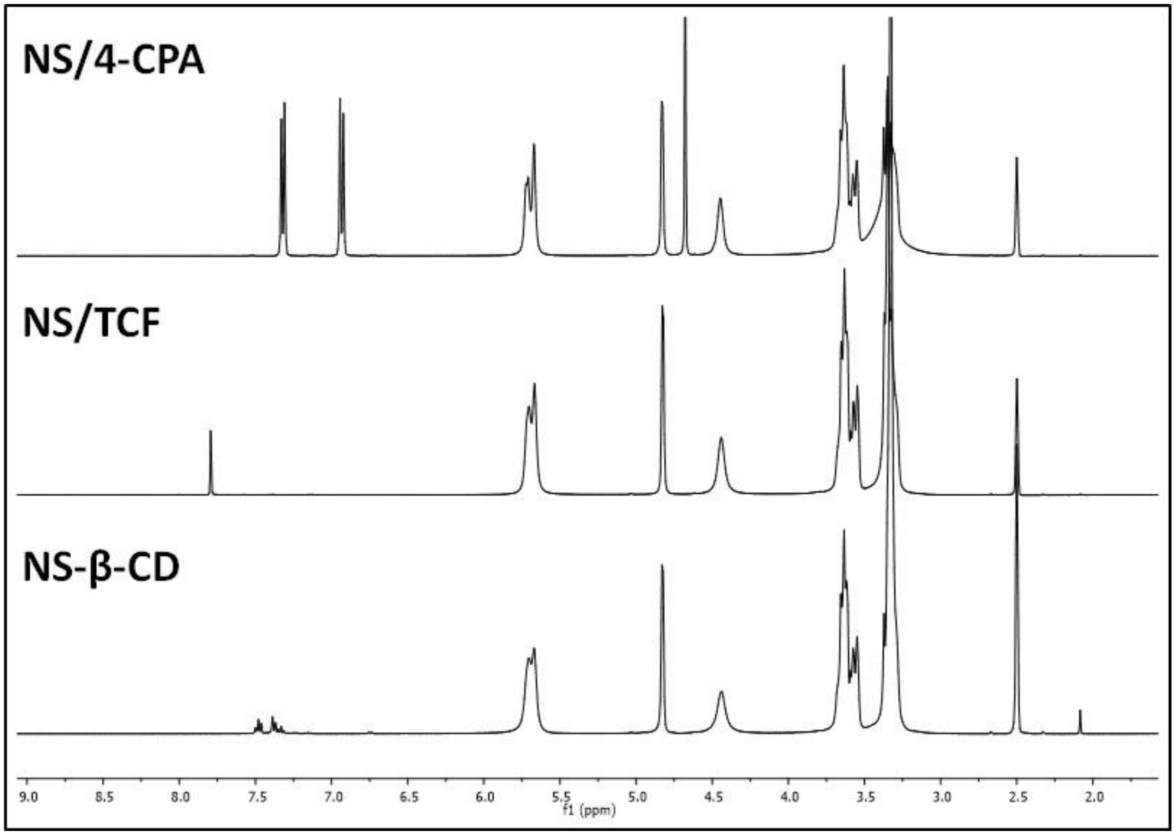

Figure S7. **^{1}H-NMR spectra of NS, NS/4-CPA and NS/TCF (400 MHz, DMSO-*d*[6])**

ZFC and FC of bare magnetite are shown in figure S8. As temperature rises from 10 to 300 K, ZCF magnetization increases and then tends to decrease, reaching its maximum at 150 K. FC magnetization gradually decreased as the temperature increased. Differences between ZFC and FC magnetization could be caused by magnetic anisotropy.

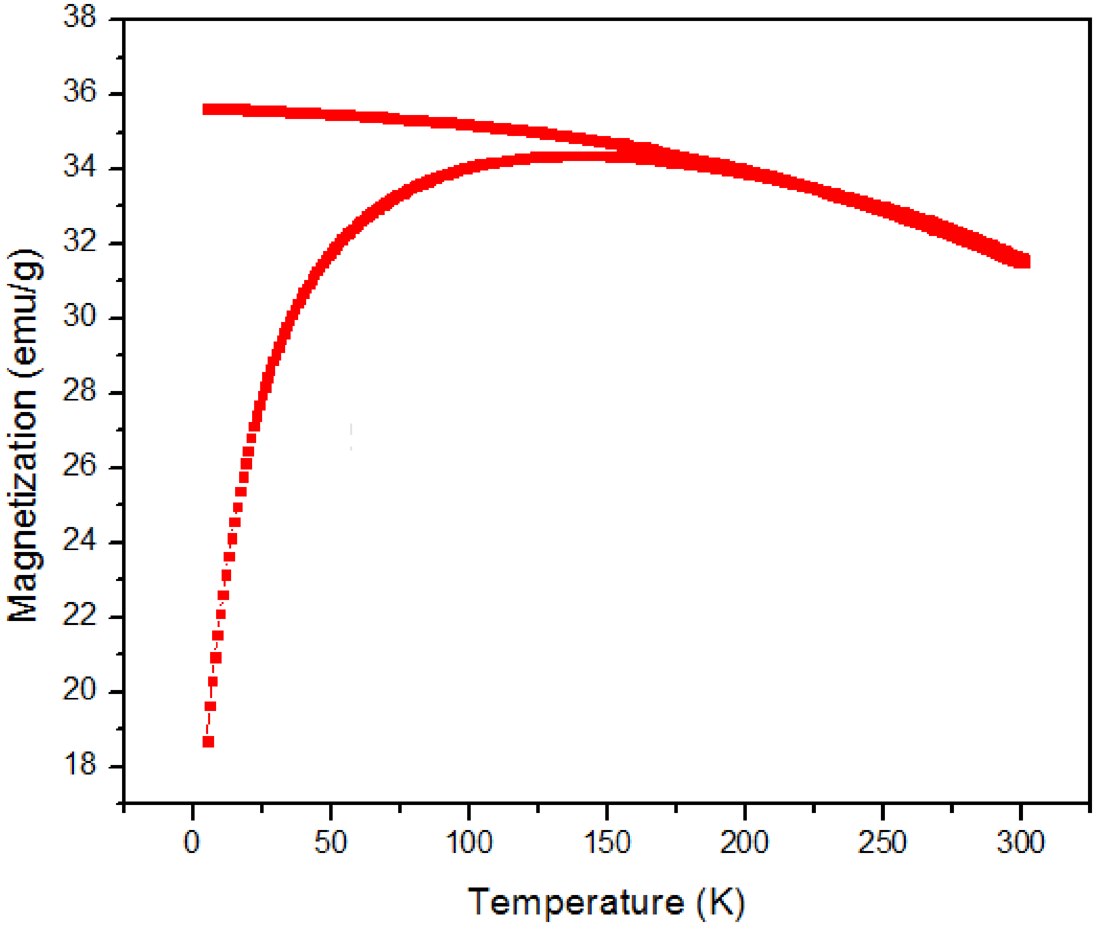

Figure S8. ZFC/FC analysis for magnetite NPs

Hydrodynamic radius, polydispersity index and Z-Potential for Magnetite nanoparticles are shown on figure S9. DLS analysis showed a hydrodynamic size for the magnetite sample of 98 nm with a PDI of 0,164. Z- Potential analysis for magnetite nanoparticles showed a value of -42, 6 mV.

			Size (d.nm):	% Intensity:	St Dev (d.nm):
Z-Average (d.nm):	98,83	Peak 1:	120,4	100,0	56,60
PdI:	0,164	Peak 2:	0,000	0,0	0,000
Intercept:	0,947	Peak 3:	0,000	0,0	0,000
Result quality :	Good				

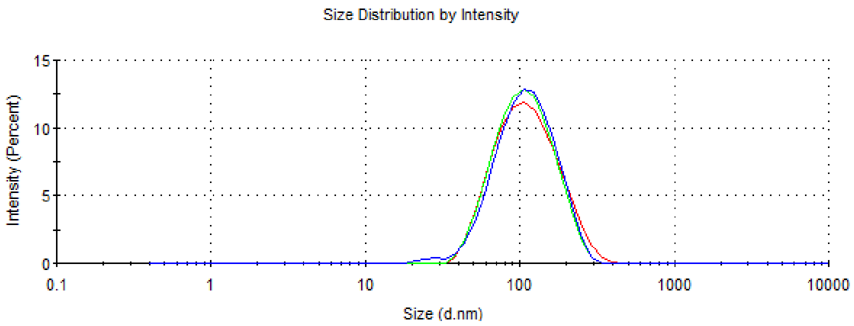

			Mean (mV)	Area (%)	St Dev (mV)
Zeta Potential (mV):	-42,6	Peak 1:	-41,3	94,6	7,95
Zeta Deviation (mV):	9,83	Peak 2:	-69,0	5,4	4,47
Conductivity (mS/cm):	0,106	Peak 3:	0,00	0,0	0,00

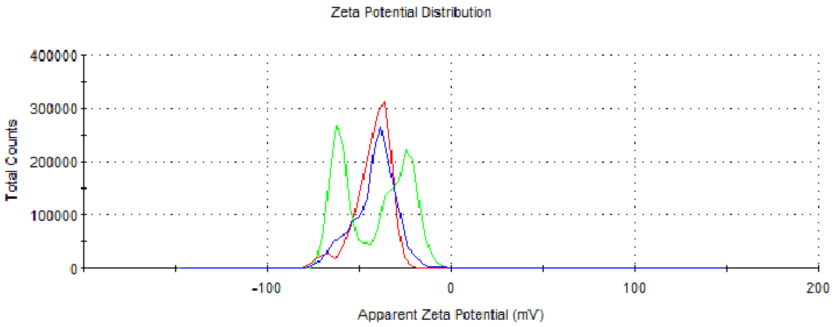

Figure S9. DLS and Z-Potential analysis for magnetite nanoparticles

TGA analysis on figure S10 shows that Magnetite nanoparticles degradation occurs on multiple steps. Degradation of Fe_3O_4 starts at 120 °C which could be due to residual moisture on the sample. There is a second peak at 320 °C where maximum mass loss of the sample takes place. Another derivative peak appears at the range of 580 – 630 °C that could be attributed to Fe_3O_4 to FeO phase transition.

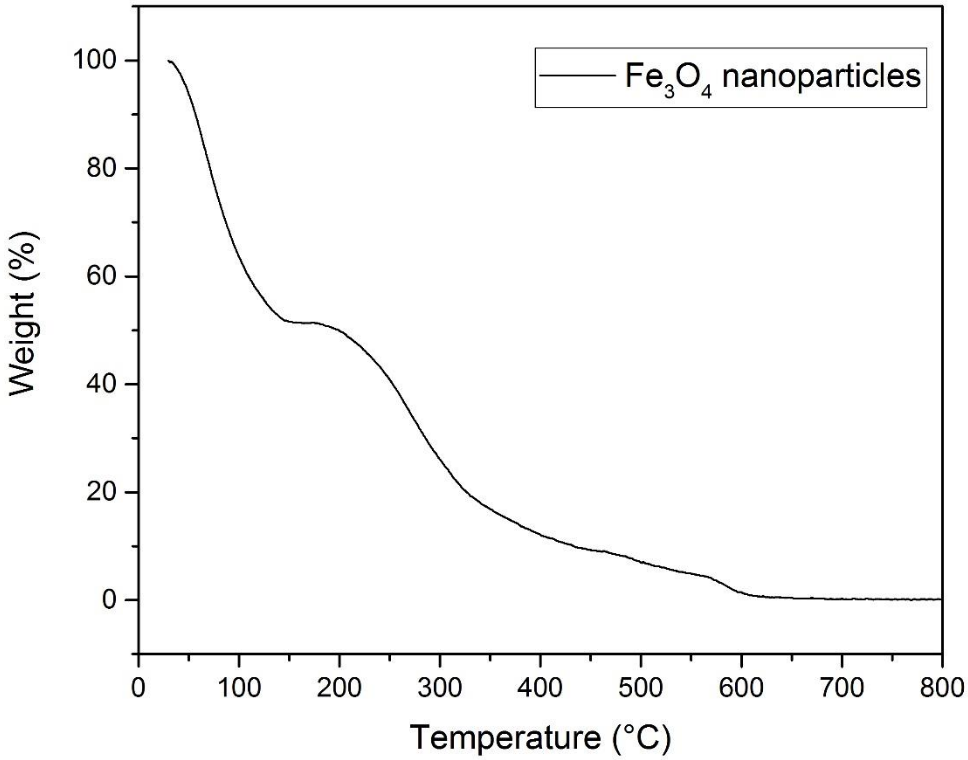

Figure S10. TGA analysis for magnetite nanoparticles

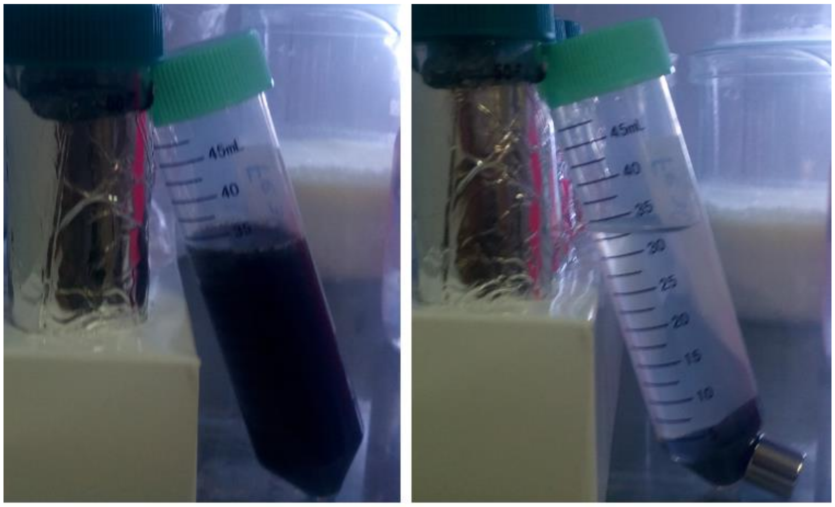

Figure S11. Response of magnetite nanoparticles to an external magnetic field.

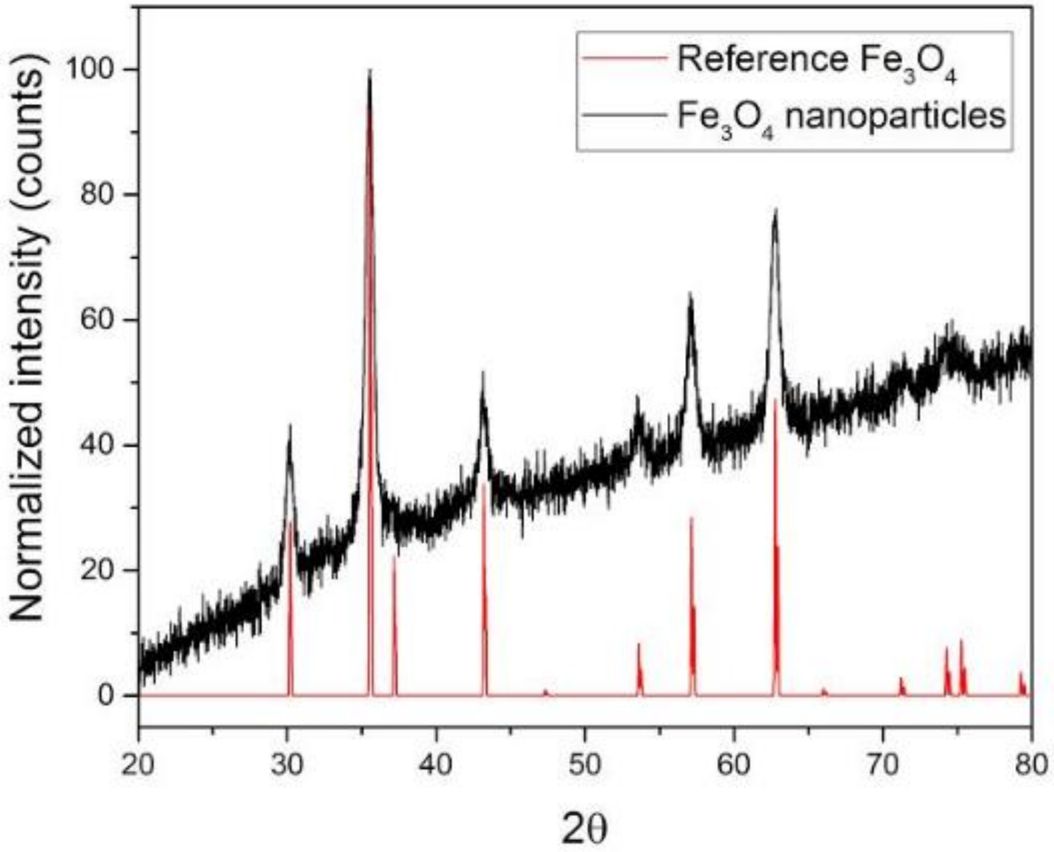

Figure S12. Comparison of magnetite XRPD analysis with standard data (obtained from http://rruff.info/magnetite/R061111, visited on 20-08-2018).

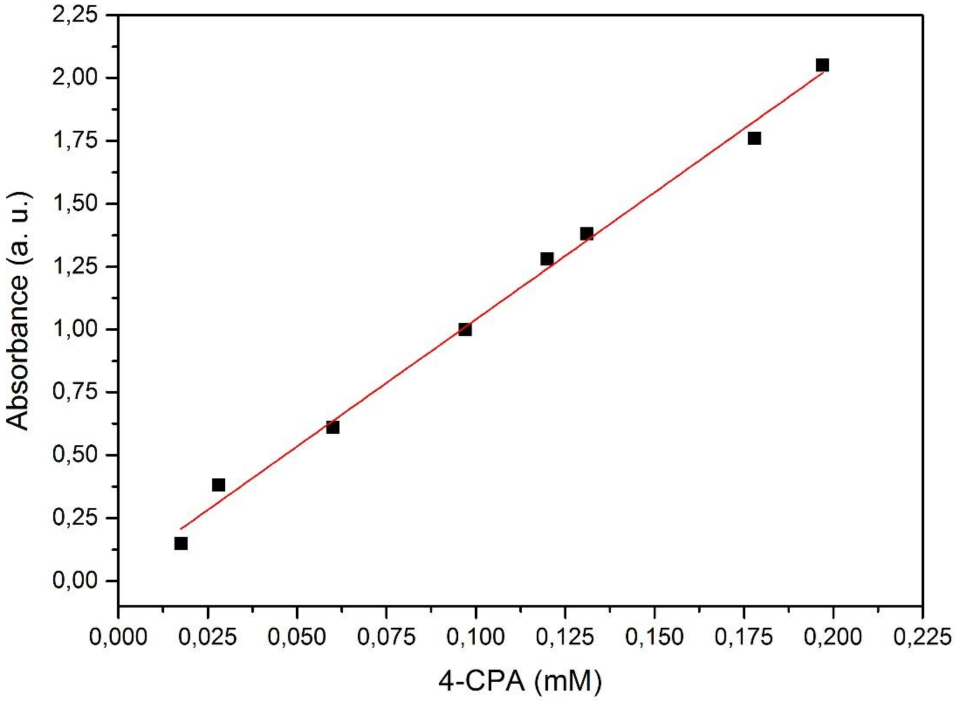

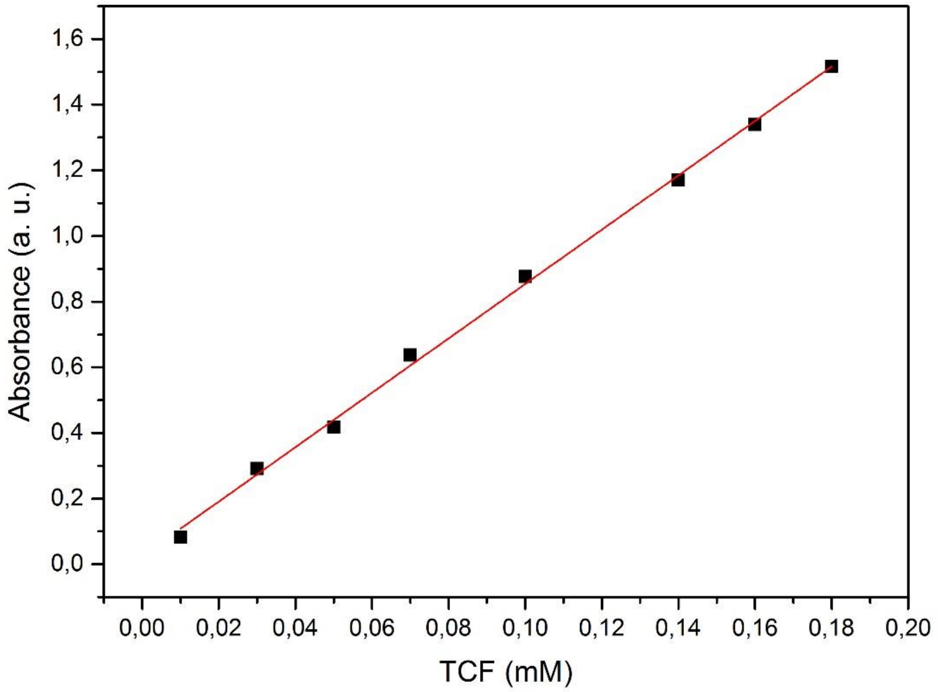

Figure S13. Calibration curves for 4-CPA and TCF
